# Supplementary material for: Are carbon emissions trading and green financial instruments synergistic? -Comprehensive quantitative research based on content analysis
Source: PLoS One. 2024 Mar 7;19(3):e0298601. doi: 10.1371/journal.pone.0298601 (PMC10919601; doi:10.1371/journal.pone.0298601)
Supplement: S1 Data — (ZIP) [file pone.0298601.s001.zip › Data/地级市Z_F工作报告.zip!/╣π╢1⁄2/╢1⁄2▌╕╩╨/└┤╫╘╣1⁄2╓┌║┼í╛┬φ┐╦╩2╛▌═°í┐_2010.docx]

各位代表：

        现在，我代表市人民政府向大会作政府工作报告，请予审议，并请政协各位委员和其他列席人员提出意见。

2009年工作回顾

        过去一年，是新世纪以来东莞经济社会发展最为困难的一年，也是我们砥砺奋进、经受严峻考验的一年。面对罕见国际金融危机带来的严重冲击，我们在上级和市委的坚强领导下，以科学发展观为指导，以实施《珠江三角洲地区改革发展规划纲要》为动力，认真落实“三促进一保持”的工作要求，加快推进经济社会双转型，着力保增长、扩内需、调结构、强管理、惠民生，在逆境中实现了新的发展。预计全市生产总值3760亿元，按可比价计算比上年增长5.3%，人均生产总值56500元，增长10%。来源于东莞的财政收入628亿元，增长4.5%，其中市财政一般预算收入231亿元，增长10.5%。城市居民人均可支配收入33045元，农村居民人均纯收入13061元，分别增长9.1%和6%。金融机构本外币各项存款余额5095亿元，比年初增长14.3%。三大产业比例由上年底的0.3∶52.8∶46.9调整为0.3∶47.8∶51.9。东莞先后获得全国文明城市、全国社会治安综合治理优秀市、全国民族团结进步模范集体等荣誉。创建国家环保模范城市顺利通过国家技术评估。

        ——这一年，我们千方百计保增长，实现了逆势回升。把保增长作为首要任务，全面实施并不断完善应对危机的一揽子计划，成功遏制了经济下滑势头，推动了稳步回升。着力帮扶企业共渡难关。实施“六个10亿元”帮扶计划，深入走访企业，帮助解决实际困难，稳定企业扎根发展。积极开展跨境贸易人民币结算试点和知识产权质押融资试点工作，出台已建房屋补办产权办法，以贴息和担保方式帮助企业融资，推动金融机构为1430家企业累计发放贷款548亿元；推广“两仓合一”保税物流模式，降低企业物流成本，建成保税仓和出口监管仓34个，东莞保税物流中心投入运营。有效增强了社会信心，企业经营逐步好转。全市外资企业关停外迁宗数减少24%。着力扩大投资刺激消费。实施五年7300亿元的投资计划，加大政府投资力度，积极引导社会投资。完成市镇两级土地规划修编，出台新的征地拆迁补偿标准；加大重点项目统筹推进力度，实行领导挂钩督导和审批绿色通道制度，对财政投资项目实行并联审批，500万元以下项目招投标权限下放镇街。开展“欢乐消费年”系列活动，实施家电下乡等八大商贸促进工程，推进国民旅游休闲计划，刺激居民消费。全社会固定资产投资1094亿元，增长13%；社会消费品零售总额956亿元，扣除物价因素影响实际增长19.7%，成为拉动经济增长的重要动力。着力鼓励出口拓展内销。出台16项出口优惠措施，协同海关提高口岸通关效率，建立出口300强企业外贸快速通道，全市出口551.7亿美元，降幅比年初收窄16.1个百分点。积极引导和帮助企业开拓国内市场，举办大中型展会48场，组织莞货促销活动24场，资助企业参加国内外大型展会50场，推动企业与销售商家、电子商务平台对接。全市规模以上工业产品内销产值2710亿元，占工业销售总产值的46%，比上年提高4.6个百分点。

        ——这一年，我们坚定不移调结构，加快了转型步伐。总结试点经验，出台“1+26”政策文件，加大结构调整力度，有效推动了三大产业结构、内外源型经济结构、企业投资结构、经营模式、加工贸易企业形态优化，以及人均经济指标、自主创新能力、节能减排水平提升。以加工贸易转型优化存量。积极搭建服务平台，完善就地不停产转型的操作办法，帮助339家来料加工企业成功转为三资或民营企业。用好加工贸易转型升级专项资金，激励企业加强技术研发和品牌经营，全市新设立外资企业研发机构84家；拥有自主品牌的加工贸易企业900家，增长15%。加工贸易合同电子审批实现全覆盖。以加强园区招商提升增量。依托市镇工业园区，调整引资目录，实行招商奖励，精心组织赴欧日韩、港台、广州、杭州等地招商推介。全市实际吸收外资26亿美元、内资122亿元，分别增长6%和42.4%。松山湖项目引进和投产步伐加快，工业产值、税收分别增长43%和40%；虎门港成功开辟首条国际班轮航线，获批深水泊位项目共21个，其中6个正式投产运营；东莞生态园完成土地征收83.4%，启动重点工程30项；长安新区用海申报、规划环评、港区总体规划等通过专家审查。以推进自主创新提高质量。完善科技东莞配套政策，积极培育“两自”企业，新增国家高新技术企业90家、省民营科技企业89家；新增省级以上名牌名标25个；专利授权量增长59.6%，总量跃居全省第二位；参与制定修订各类标准32项。加强创新平台建设，深化产学研合作，加快技术更新改造，建成省级以上各类研发和检测中心36个，新增省级产业集群升级示范区2个，承接省级以上科技项目313个，获批省部院市产学研合作项目99个，全市工业更新改造投资154亿元，增长1.5倍。支持科技企业融资，设立5000万元科技贷款风险准备金。整合出台扶持民营经济22条措施，落实排忧解难协调制度，民营企业注册资金增长13.4%；内源型经济占生产总值的62.5%，比重提高4.5个百分点。以发展第三产业强化支撑。加强现代服务业引进，推动金融、物流、会展等生产性服务业加快发展。东莞农信社改制为农村商业银行，新增银行和融资担保机构8家、上市公司1家，引进中外运等一批大型物流仓储项目，成功承办首届中国动漫博览会和广东外博会，电博会、虎门服装、厚街家具、大朗毛织、长安五金模具等品牌展会的影响力稳步扩大。出台鼓励总部经济发展的政策和实施细则，启动首批总部企业认定工作。第三产业增加值占生产总值的比重提高5个百分点。以落实节能减排严格约束。强化责任落实，加强重点耗能企业监管，开展全民节能行动，推进企业清洁生产，加强土地执法监察，严守耕地保护红线。全市单位生产总值耗地、耗电分别下降3.1%和4.2%。二氧化硫和化学需氧量排放分别下降9.2%和6.8%，达到省总量减排年度要求。

        ——这一年，我们加大力度扶镇村，夯实了基层基础。把镇村作为全市发展和稳定的基础，市财政共支出40.9亿元，大力支持镇村建设和发展。加大经济扶持管理力度。完善财政激励政策，欠发达镇超收分成由30%提高到40%，继续实施贷款贴息等帮扶政策，市财政共贴息2.2亿元，帮助欠发达镇村累计获得贷款49亿元，对285个村（社区）补助公共管理经费2.35亿元。加强镇村收支和资产监管，出台农村干部违反集体资产管理行为责任追究办法，开展重大事项审查和村级绩效审计，强化农村集体土地款、借贷款与经济合同管理，严格控制集体开支和福利分红。32个镇街可支配收入221.5亿元，增长15.9%；村组两级净资产890.2亿元，增长3.5%。加大基础设施建设力度。“三院一中心”等28项市属重点工程竣工，市轨道交通R2线起点站、东莞大道延长线、东莞篮球中心等38项动工。环城路全线贯通，莞深高速三期、虎岗高速顺利通车，建成镇村联网路14条、大型输变电工程17项、水利防灾减灾工程123宗。整合村级水厂16家，改造老化水管547公里，新增LNG管网163公里，整治市区内涝点11个。加大城乡环境整治力度。调整优化生态控制线和饮用水源保护区，实施生态补偿，促进生态保护。加快环保基础设施建设，累计建成污水处理工程32项、截污主干管网工程18项，医疗废物处理中心投入营运。扎实推进运河综合整治，运河水质明显改善。加强环境执法监察和污染企业监管，积极治理养殖业污染、水库污染、油气污染和机动车尾气排放。出台“三旧”改造办法和实施细则，深入推进“五整治”，加强城管综合执法，狠抓城市“六乱”治理，进一步提升了市容环境水平。加大现代农业发展力度。加快农业产业园建设，已批10个中6个完成了主要基础设施建设，2个被认定为省级园区。推动农业产业化、规模化、标准化经营，新增省、市级农业龙头企业各2家。启动集体林权制度改革试点工作。

        ——这一年，我们多管齐下强管理，维护了安全稳定。把综治维稳作为第一责任，着力加强社会管理，妥善化解矛盾纠纷，保障了安定局面。强化社会治安综合治理。加强巡逻防控，整治重点区域，保持严打高压态势，突出打击“两抢一盗”、拐卖儿童等犯罪行为，开展禁毒禁赌扫黄专项行动。推进科技强警，建设视频监控联网平台，扩大视频监控覆盖面，全市在用各类视频监控点12.8万个。新建平安社区200个。全市接报违法犯罪宗数下降4.3%，刑事案件破案宗数增长60.4%。强化安全隐患整治。加快安全生产应急救援指挥平台建设，划定了21个应急避险场所，全市所有村（社区）都成立了安全办。深入开展消防隐患、危险化学品、食品药品和建筑安全等专项整治行动。加强农产品检疫检测，建成食品安全样板市场38个。强化交通安全管理，禁止摩托车在环城路以内行驶，大力查处酒后驾驶等违章行为。全市发生各类事故起数、死亡人数分别下降6.4%和7.3%。强化矛盾纠纷调解。新建镇街综治信访维稳中心22个，村（社区）、企业综治工作站（室）317个。扎实开展“基层大接访”和“信访积案化解年”活动，全市受理群众信访总量下降39.4%，群体性事件下降44%。完善企业倒闭风险预警机制，严厉打击欠薪逃匿行为，为9.3万名工人追回工资1.2亿元。

        ——这一年，我们高度重视惠民生，增进了社会和谐。把改善民生作为政府工作的出发点和落脚点，全面发展社会事业，基本完成向市民承诺的十件实事，市财政用于民生事业支出102.8亿元。办好教育实事。以积分制方式新接受1.39万名新莞人子女入读公办学校。取消义务教育公办学校非本市户籍借读生书杂费，免收金额2.3亿元。切实解决中小学代课教师待遇问题。出台扶持民办教育发展政策。推进高中阶段学校布局调整，实施中小学校舍安全工程，新建学生宿舍10座，增加床位近万个。落实“六优先”政策，促进各级各类教育优质均衡发展，每万户籍人口升大学人数排名全省第一。繁荣文体事业。实施“提升城市文明十大行动”和“文化惠民工程”，新建图书馆和博物馆12个、农家书屋和妇女书屋142个，免费开放了一批文化馆所。开展庆祝新中国成立六十周年活动，举办省音乐舞蹈花会和东莞音乐剧节，启动历史人文创作工程，莞城“文化周末”活动荣获文化部创新奖，12个文艺精品获得全国全省重大奖项。大力发展群众体育和竞技体育，成功承办举办第四届全国妇女健身活动展示大赛、市第七届运动会等大型赛事20余次，莞籍运动员在第十二届全运会上取得7项冠军，获得省第二届体育大会团体总分第四名。完善社会保障体系。统筹农居民与职工养老保险制度，两次提高医保待遇，将大中院校学生纳入基本医保范围。增加生育医疗待遇，整合医疗和生育保险。完善低保制度，提高“五保户”供养标准，开展居家养老服务试点。为23800多名户籍困难残疾人提供生活专项补助和服务。加快社会工作发展，扶持社工机构建设。解决2658户困难家庭的住房问题，建成经济适用房1454套，完善住房公积金管理，制定改善新莞人居住条件指导意见。全面发展卫生、劳动就业、计划生育等事业。完善社区卫生服务体系建设，积极防控甲型H1N1流感，开展职业卫生和生活饮用水专项整治。帮助户籍大中专毕业生和零就业家庭就业，完成户籍劳动力资助培训和新莞人技能提升培训6.7万人次。城镇登记失业率1.7%。建立37个新型社区，解决人才落户难问题。大力发展公共交通，投放城市小巴，设立学生临时专线，新增公交运力302辆、线路48条。提升人口计生服务管理水平，人口自然增长率6.31‰。完成第二次全国经济普查。国防人防、科普法普、外事侨务、工青妇幼、民族宗教、统计审计、气象测报、档案方志、打私等工作扎实推进。

        ——这一年，我们改进作风抓落实，提高了政府效能。把加强政府自身建设摆在突出位置，以更务实高效的作风抓好工作落实。推动相关改革。实施政府机构改革，市政府部门从37个整合为32个。以石龙、塘厦为试点推进扩权强镇，下放管理权限248项。完成第三轮行政审批制度改革，取消审批事项85项。恢复驻京联络处，密切与中央机构和企业的联系。弘扬务实作风。加强督查督办，推广现场办公、联合会审等工作方式，帮助基层和企业解决实际问题。出台法治政府建设意见和重大行政决策程序规定，推进政务公开，进一步规范政府行为。落实廉政责任，开展机关作风暗访和行业纠风行动，加强电子纪检监察系统应用，职能部门审批业务提前办结率达98.8%。全年办理人大代表和政协委员的建议提案489件。厉行勤俭节约。加强财政资金绩效评价和审计，提高资金使用效益。严格控制楼堂馆所等非生产性项目投资，严格控制一般性行政经费支出，市直机关行政经费缩减5.3亿元，由财政出资或政府主办的晚会、展览、庆典、论坛活动经费减少47.5%。

        ——这一年，我们落实《纲要》谋合作，促进了区域协调。深入实施珠三角规划纲要，积极加强区域合作，努力开创区域协调发展新局面。扎实推进深莞惠一体化。建立三市联席会议制度、政府工作协调机制、专责小组协调推进机制，召开党政主要领导联席会议3次，签署框架协议文件13份，确定并积极推动近期合作的25项重点事项。扎实推进莞港台合作。加强与港台地区在帮扶企业、招商推介、内外贸易等方面的合作，成立莞港生产力促进中心，促成台湾10大产业服务机构在莞设点，创办松山湖台湾高科技园。扎实推进双转移战略。制定产业转移规划和实施方案。市财政投入1亿元加快建设莞韶、莞惠产业转移工业园。接收我省东西北地区近50万劳动力就业。扎实推进对口援建扶贫。按照三年任务两年完成的要求，加快汶川映秀镇灾后对口援建，总投资近10亿元的33项“交钥匙”工程和25项资金补助项目已完成总工程量的50%。全市捐助台湾同胞抗灾重建1.4亿元，援助广西河池、西藏林芝等对口帮扶地区财物5742万元。

        各位代表！

        回顾一年来同舟共济的拼搏之路，我们深感成绩凝聚汗水，团结就是力量。我市在极其困难的形势下保持了经济社会的平稳发展，离不开上级和市委的正确领导，离不开各级各部门的精诚协作，离不开市人大、市政协、各民主党派及社会各界的监督支持，离不开广大投资者的信赖、坚守和勤奋经营，离不开全体市民和驻莞部队的共同努力，离不开港澳台同胞、海外侨胞以及国际友人的关心帮助。在此，我们表示衷心的感谢！

        回顾一年来逆势前行的突围之路，我们深感信心异常可贵，坚持就是胜利。困难是常有的，也是暂时的，战胜困难的过程，就是推动改革发展大步向前的过程。东莞能够成就今天的城市地位，靠的就是敢为人先，务实奋进，靠的就是不畏险阻，艰苦奋斗，靠的就是坚定信念，永不言败。

        回顾一年来化危为机的进取之路，我们深感科学发展制胜，转型才有出路。实践证明，只有坚定不移地落实科学发展观，坚定不移地推进经济社会双转型，才能有效转变发展方式，提高核心竞争能力和抗御风险能力，才能推动东莞经济社会不断取得新的发展。

        回顾一年来攻坚克难的奋斗之路，我们深感问题不容忽视，忧患才能兴市。当前东莞仍存在不少问题和隐忧：经济回升基础尚不牢固。主要指标增长与年度目标有一定差距，外贸出口尚未完全恢复，合同利用外资降幅较大，企业经营还有不少困难。转变发展方式任重道远。引进培育大项目等工作亟需加力，自主创新能力和抗御风险能力有待进一步提高，污染整治的成效仍未充分显现。社会管理工作仍然繁重。社会不稳定因素依然较多，治安形势仍未实现根本好转，群体性事件、安全和消防事故时有发生。我们必须高度清醒，以强烈的责任感和紧迫感，积极寻求破解之策。

2010年工作安排

        今年是实施“十一五”规划的最后一年，做好全年各项工作，对夺取应对国际金融危机全面胜利、加快经济社会转型步伐、为“十二五”规划奠定良好基础具有十分重要的意义。我们要清醒地看到，新一年的形势仍然复杂严峻，国际金融危机对东莞的影响尚未完全消除，后危机时代还会有一段比较艰难的时期，必须切实增强危机意识和风险意识，决不能有丝毫的懈怠。同时，我们也要满怀信心地看到，当前综合发展环境正在不断好转：从外部环境看，世界经济逐步显现出复苏迹象，外部需求有所回升，我市外贸出口压力将得到一定程度的缓解；不少跨国公司正在酝酿产业的重新布局，有利于我市开展招商引资。从国内环境看，中央和地方一系列刺激经济政策日益见效，中央继续实施积极的财政政策和适度宽松的货币政策，珠三角规划纲要深入实施，有利于我市开拓国内市场，深化区域合作。从我市实际看，经过一段调整适应期，经济企稳向好的势头明显，全市上下应对危机的信心和能力不断强化，推动转型发展的积极性和主动性进一步增强。只要我们审时度势，坚定信心，趋利避害，奋发有为，就一定能够实现新的更大的发展。

        综合考虑各方面因素，全市经济社会发展的目标是：生产总值增长8%；市财政一般预算收入增长6%；固定资产投资和社会消费品零售总额增长14%；外贸出口增长5%；城市居民人均可支配收入和农民人均纯收入增长7%；单位生产总值能耗下降4.4%；城镇登记失业率、人口自然增长率分别控制在2.5%和7‰以内。

        政府工作的总体要求是：以科学发展观为指导，全面贯彻党的十七届四中全会、中央经济工作会议、胡锦涛总书记视察广东重要讲话、省委十届六次全会和市委十二届六次全会精神，深入实施珠三角规划纲要，落实“五个更加注重”和“三促进一保持”要求，突出以转变经济发展方式为核心，继续稳增长，加力调结构，积极优环境，锐意抓改革，更加重民生，全面促和谐，坚定不移地推动全市经济社会在平稳发展的基础上加快转型，全面完成“十一五”规划各项目标任务，努力建设以信息产业为特色的现代制造业名城、适宜创新创业安居乐业的生态城市、珠三角新兴物流城市，当好推动科学发展、促进社会和谐的排头兵。

        具体抓好以下八个方面的工作：

        一、坚持多策并举，巩固经济回升基础

        珍惜来之不易的回暖局面，把扩大内需作为战略基点，继续实施刺激经济增长的一系列政策措施，不断巩固和增强回升向好的势头。

        完善落实扶企政策。加强政策宣讲辅导，完善管理办法和操作指引，切实发挥好科技东莞、创业东莞、转型升级、支持融资等多个专项资金的作用。融资贴息、社保金缓交等政策延期一年，扩大政策普惠面，加快补办土地使用证和房屋产权证，帮助企业盘活资产。积极推进跨境贸易人民币结算试点工作。加强劳务协作，完善用工平台，帮助企业解决招工难题。

        加快投资项目建设。全面加快重点基础设施和优质产业项目建设，将政府投资5000万元、社会投资2亿元以上的项目列为重点项目，给予纳入审批绿色通道、减收城市基础设施配套费等支持。结合产业导向和引资目录，制定优质产业项目认定标准，做到用地指标优先安排、审批事项优先办理。完善和落实领导挂钩督导等制度，定期协调解决实际问题，加大投资拉动力度。

        积极开拓内外市场。落实激励政策，帮助企业抢抓订单、稳定出口，积极开拓东盟等新兴海外市场，优化外贸出口结构。加力资助企业拓展内销，推广内销“集中申报”模式，加快保税物流和口岸大通关建设，积极举办形式多样的莞货展销活动，认真筹办省第二届外博会，资助企业参加各类大型展会，组建东莞工厂直销中心，推动生产企业与大型商家、电子商务平台对接。积极举办各类商贸促销活动，加大家电和汽车下乡、以旧换新等政策的宣传力度，发展和完善消费信贷，有效刺激和扩大居民消费。

        二、加快结构调整，构建现代产业体系

        坚定不移地把产业结构调整作为核心任务和工作主线，加大典型示范和推进力度，有效转变经济发展方式。

        加快打造三大经济带。着力打造以松山湖为龙头的园区经济带。完善松山湖各项功能配套，推进台湾高科技园和IT研发园建设，加速集聚高端产业和人才；同步加强东莞生态园的基建与招商工作，完成主要路网设施建设，力争在项目引进上有所突破；推进长安新区规划报批，基本完成收地任务，尽快转入实质性建设阶段。着力打造以虎门港为依托的物流经济带，积极寻求战略合作，加速码头泊位建设运营，研究出台港口物流优惠政策，提升港口集散能力，推动早日繁荣；加强西大坦物流园和立沙岛石化园的招商，积极引进精细化工和装备制造等产业。着力打造以主城区为核心的商贸经济带，推进中央商务区、商贸区和休闲区的规划建设，强化主城区的辐射带动功能。

        加快推进加工贸易和传统产业转型升级。完善专项资金使用办法，优化一站式服务，重点跟进资金技术密集型来料加工企业，推动其不停产转三资或民营企业，在拓展内销、技术改造、设立研发机构和地区总部等方面取得实效。加强与港台专业机构合作，帮助企业提升技术和管理水平。以信息化和高新技术改造提升传统产业，推动五金模具、服装、毛织、家具等产业集群优化升级。

加快引进培育优质项目和龙头企业。加强已有产业分析和投资动向研究，落实重大及关键项目引资奖励措施。加大城市营销力度，充分利用现有企业资源，对重点企业开展登门招商，对日韩台等地区实行驻点招商，积极引进国内外大型企业和行业龙头企业，引进新兴战略性产业、先进制造业及其配套缺失项目。着力扶优扶强，落实24项倾斜措施，突出扶持市选定的521家重点工业企业。力争用3年时间形成一批年主营业务收入超十亿、百亿元的大企业。

        加快发展现代服务产业。从今年起连续5年，市财政每年安排3000万元，采用竞争性招标方式资助信息服务业项目。出台建设珠三角新兴物流城市的实施意见，认定十大连锁企业、十大物流项目、十大专业市场和十大展会，从政策和资金上重点扶持。加强对同类型专业市场及展会的规划整合。大力发展工业设计、金融、旅游、中介等产业，稳步发展总部经济。把文化产业作为支柱产业来扶持，制订发展规划和配套政策。认真筹办第二届动漫博览会，引进培育文化创意产业基地，推动现代传媒、演艺娱乐、印刷等文化产业集群发展。

        加快落实节能降耗减排。强化污染企业监管，严把产业项目准入关，加快7个环保专业基地建设。务实发展低碳经济，落实重点耗能企业节能计划，扶持发展节能服务机构，鼓励企业开展清洁生产。推广清洁能源、节能建筑、绿色照明，实施LED路灯应用示范工程，深化机动车尾气和加油站油气污染治理。严格落实责任，确保完成“十一五”节能减排任务。

        三、强化自主创新，提高核心竞争能力

        把自主创新作为引领东莞未来发展的核心推动力，深入实施“科技东莞”工程，积极培育“两自”企业，大力发展民营经济，不断提升自主发展能力。

        推动企业研发和品牌经营。落实专项扶持资金，鼓励企业加大技改投入，设立研发机构，承接参与国家和省市重大科技专项，争取新建一批省级以上工程技术中心，推动一批企业成为省级工业设计示范基地。做好首届市政府质量奖评审工作，鼓励企业参与各级各类标准的制定和修订。深入实施名牌带动战略，支持企业争创名牌名标，加强知识产权和品牌保护。

        推动科技平台建设和管理。推进国家设计中心试点工作，推动散裂中子源项目动工，加快中山大学研究院、国家消费品重点实验室等公共平台建设，新建鞋业、模具等行业技术平台，组建太阳能、智能玩具等省部产学研创新联盟，引导镇街以“民办公助”方式建设科技企业孵化平台。加强分类指导和管理，促进现有科技平台提高运营水平和效益。办好科技合作周等活动，推动高等院校与企业的产学研合作，积极谋求关键领域和关键技术突破，加快科研成果转化应用。完善和落实人才政策，争取在科技创新团队和领军人才的引进培育方面有所突破。

        推动民营经济做大做强。进一步放宽市场准入，落实扶持民营经济发展22条措施，突出帮扶省百强民营企业和市50强民营企业。实行核发筹建执照等办法，推动规模较大的民营企业加快落户。制定相关激励政策，引导民间资本更多地进入先进制造业领域。对民营企业的大额技改投资贷款给予贴息，对技术开发和转让的相关税费给予减免，完善扶持民营企业的政府采购政策。充分发挥商会行会作用，加强对民营企业家的培训，鼓励企业实行强强联合、并购重组，实现规模化发展。

        推动科技金融更紧密结合。全面落实金融业发展规划，加快市金融商务区和松山湖金融改革创新服务区建设，积极组建外商和台商信用担保公司，推动设立村镇银行，稳步发展融资、租赁机构。做好后备企业改制上市培育工作，力争新增上市企业2—3家。积极开展省首批科技金融试点市工作，制定市联动科技贷款风险准备金使用办法，推动知识产权质押融资，发挥创业投资和风险投资作用，引导更多资金投向科技创新领域。

        四、着力帮扶镇村，以强镇促进强市

        镇村是东莞的发展之基、特色所在、惠民所依，强镇才能强市。必须坚持不懈地扶持镇村，筑牢全市发展基础。

        加强政策资金扶持，加大转移支付力度。全年市财政安排49.4亿元扶持镇村发展。落实好财政超收分成、公共管理支出补助、生态补偿、结对帮扶等政策措施。继续按一定比例分担联网公路、水利工程等基础建设费用，村际联网路补贴政策期限延长一年。对镇村集体已建项目补办手续给予费用优惠。突出帮扶欠发达镇村加快发展，继续实施扶贫贷款贴息政策，适当放宽贷款使用范围。引导欠发达村用好市免息借款，不断增强造血功能。

        加强土地统筹开发，转变镇村发展模式。强化镇级统筹发展的主导作用，鼓励各镇街以租赁、入股、征收等多种形式，加强对土地的统筹利用，实行统一规划、统一建设、统一招商，不断完善镇村“统筹开发、利益共享”的发展模式。探索建立相关激励机制，对镇街涉及土地统筹的贷款给予贴息，重大项目的税收给予所在村一定比例的返还。

        加强集体资产监管，力促增收节支减债。制定村组年度收支计划，合理控制资产负债率。积极扶持集体增收项目。逐步减轻村组社会管理负担。严格落实重大事项审查、土地款管理、合同管理等制度，加强农村集体经济审计和责任追究。规范农村干部薪酬管理。继续严控一般性支出，严控贱卖集体资产，严控超支福利分红。

        坚持生态高效方向，扶持现代农业发展。按照“强化规划、突出产业、标准适度”的原则，逐步把全市符合条件的农业用地纳入园区范围。推进标准化基本农田建设，承接优质高产的农业项目。积极培育农业产业化组织，推动专业合作社发展，推广现代农业机械和技术，探索土地流转和规模经营的有效方式，推动农业产业园建设速度加快、效益提高。

        五、统筹规划建设，提升城乡环境水平

        坚持组团发展方向和环境取胜战略，不断强化城市功能，改善城乡环境，建设生态宜居城市。

        加速推进城市基础设施建设。配合推进穗莞深、穗莞惠城际轨道交通工程，全面启动市轨道交通R2线建设，推进石龙火车新站工程。加快完善“一环六纵六横三连”的高快速路网，建成虎岗高速虎门港支线一期、广深高速石鼓连接线，推进广深沿江高速、从莞高速、博深高速、深圳外环高速东莞段以及东莞大道延长线、环莞快速路二期、松山湖大道至常平连接线、高埗连接市区梨川大桥等建设。加快中麻公路、石大公路、省道S120石排至桥头段、S256南城至虎门段、S357谢岗段、S358长安至虎门段等改造工程。加快水、电、气网建设，完成东江与水库联网一期工程，建成水利防灾减灾工程126宗，改造供水管网1300公里；建成110千伏及以上输变电工程15项；新建天然气管网131公里。加快打造“无线城市”，推进电信网、广电网、互联网三网融合。

        加速推进城市功能配套建设。加快大市区建设，抓紧完成广电中心、篮球中心、市中医院新院、市民艺术中心、工人文化宫、市廉租房和经济适用房等项目建设，增强城市功能和辐射带动作用。加快各镇中心区建设，按照中小城市的标准完善公共设施，着力打造若干个各具特色的标志性片区。加快成熟社区建设，制定量化标准，完善公交、商业、休闲等功能配套，力争年内每个镇打造出2—3个成熟社区，市区四个街道成熟社区覆盖率达到40%。

        加速推进旧城旧村旧厂改造。把“三旧”改造作为优化城市布局、改善城乡面貌、促进经济发展的重要抓手强力推进。设立市财政连续五年、每年10亿元的专项资金，落实减免市属税费、简化供地方式、优先用地指标、加快项目审批、返还出让收益等优惠政策。抓好分类典型示范，通过开发商业区和商住楼、改建现代化工业厂房、联建农民公寓、保护历史村落、建设主题公园等多种模式推进改造。各镇街年内完成改造专项规划，至少启动一个成片改造和若干个单宗改造项目。

        加速推进城乡环境综合整治。落实生态绿线规划，严格保护农田山林绿地。按照“一年基本建成、两年全部到位、三年成熟完善”的要求，扎实推进珠三角绿道网建设。加快黄旗山城市公园的建设改造，完善市植物园和银瓶山、大岭山、大屏嶂森林公园的设施配套。鼓励各镇村因地制宜建设乡村公园。坚持广泛植树、见缝插绿，按照新区不低于30%、旧城改造区不低于25%的面积标准配套绿地。加大运河综合整治力度，推进内河涌和水库污染治理。全面建成34项污水处理厂及配套管网，加强运营管理。新建4座垃圾处理厂。加大城管综合执法力度，深入整治城市“六乱”。争取成功创建国家环保模范城市。

        六、加强社会管理，切实维护安全稳定

        稳定压倒一切，必须毫不放松强化公共安全管理和矛盾排查调解，切实维护安定有序的社会局面。

        坚持不懈推动社会治安持续明显好转。保持高压态势，继续严打各类恶性犯罪和多发性犯罪，突出打击入室抢盗、公交扒窃、拐卖儿童等。深入开展打黑除恶禁毒扫黄等专项行动，大力整治治安重点区域和行业。实行有奖举报，严格责任追究，坚决清除“老虎机”和地下“六合彩”赌博行为。加大路面巡逻力度，加快视频监控网络建设，加强出租屋管理，推动社区群防群治，力争全市80%的社区（村）成为平安社区。推进公安信息化、执法规范化和队伍正规化建设。

        坚持不懈狠抓安全生产排查整治。夯实基层基础，创新监管机制，突出抓好危险化学品、特种设备、“三小”场所、人员密集场所、建筑安全等专项整治，对存在重大隐患、未能限时整改到位的场所，坚决实施关停，严防重特大事故发生。加强食品药品和农产品安全监管，创建62个食品安全样板市场，确保百姓用药安全、饮食放心。强化宣教培训，营造全社会关爱生命、关注安全的良好氛围。

        坚持不懈做好信访调解和应急管理。深入推进镇、村和企业综治信访维稳机构建设，强化矛盾纠纷排查化解，推行领导接访包案和干部下访制度。加强劳动监察执法，提高劳动仲裁处理效率，进一步健全欠薪逃匿防范机制。加快打造市综合应急平台，强化信息研判、指挥调度、辅助决策等功能，推进应急避险中心和镇街值班室建设，完善应急预案管理体系，不断提高应对和处置突发事件的能力。

        坚持不懈推进新型和谐社区建设。以社区为基本单元，完善警务区建设，落实辖区治安队、安全生产监管人员等工作职责，加强城乡社区网格化、全覆盖的管理。指导各居住小区成立业主委员会，推进物业管理进社区。年内规范完善215个城市社区的服务设施和机构队伍，逐步建立政府向社会组织购买公共服务机制，积极健全社区服务体系。深入创建省“六好”平安和谐社区，力争80%以上的城市社区达标；积极创建全国和谐社区示范单位。

        七、办好民生实事，全面发展社会事业

        坚持以人为本、惠民先行，大力发展社会民生事业，继续办好“十件实事”，不断改善群众生活，共享发展成果。

        促进充分就业、全面保障。加强就业服务，设立实名制劳动力资源库，推广“村民车间”模式，帮助户籍劳动力特别是“零就业”家庭和大学毕业生就业。实施全民技能提升储备计划，不断提高劳动力就业素质。全面深化社会养老保险体系建设，市镇村三级分期投入43.4亿元，其中今年14.5亿元，逐步提高59万名原农居民参保人员的保障水平。加快组建市社会福利中心，新建4间敬老院，向全市80岁以上老人发放政府津贴，争取一、二档镇街居家养老服务覆盖面达到20%。对低保对象实施分类保障和基本医疗救助，对特殊困难对象增发保障金。积极推进社会工作，力争专职社工达到500名。逐步提高困难残疾人补助标准，加强残疾人服务体系建设。推进住房公积金事业发展和廉租房、经济适用房建设，解决2404户困难家庭住房问题。深入开展关爱新莞人系列活动。

        促进教育质量更高、医疗卫生服务更优。坚持教育优先战略，推进各级各类教育优质均衡发展。加快高中阶段学校布局调整，新建扩建16所高中学校。推进技师学院、高技能公共实训中心和理工学校、卫校新校区等建设，加快打造职教城。探索公办中职学校学生逐步实现学费全免。支持办好东莞理工学院城市学院新校和中山大学新华学院，推进与香港理工大学联合办学，加强民办教育的扶持、引导和监管。推进市镇医院和重点特色医疗专科建设，继续完善社区卫生服务体系，实施基本药物制度，加强甲型H1N1流感防控，为群众提供优质安全、价廉便利的医疗保健服务。

        促进交通更顺畅、出行更便利。完善镇村（社区）交通分流系统，加强交通疏导和执法管理，深入推进“治摩”工作，改造3条主干公路12个交通堵塞点，整治事故多发和市镇中心区拥堵路段，努力做到安全顺畅有序。设立道路交通事故社会救助基金。大力发展公共交通，加大运力投放，优化线路设置。更新出租车1200辆，完善公交枢纽站、首末站、候车亭、出租车停靠点和交通标识、自行车道等设施，增建人行天桥和地下通道，使群众出行更加便利。

        促进文化、体育等社会事业全面发展。着力巩固全国文明城市创建成果，鼓励发展志愿服务，引导市民践行道德规范，形成良好文明风尚。完善公共文化服务体系，推进社区文化和企业文化建设，鼓励文艺精品创作，加强社会科学研究，传承文化遗产，弘扬东莞特色文化，积极创建全国历史文化名城。落实《全民健身条例》，增加健身场所和完善设施，广泛开展群众体育运动，积极承办第十六届亚运会举重赛事。科学编制“十二五”规划，开展第六次全国人口普查，加强人口计生服务管理，继续做好国防人防、双拥共建、科普法普、外事侨务、工青妇幼、民族宗教、统计审计、档案方志、打私、气象等工作。

        八、继续深化改革，激发体制机制活力

        坚持先行先试，锐意创新，大力推进管理体制机制改革，不断提升政府行政效能和公共事业活力。

        深化行政管理体制改革。全面推进政府机构改革后续工作，加快制定实施部门“三定”方案，理顺职责关系，完善运行机制，切实强化统筹管理职能。建立机构编制实名管理制度。深化户籍制度改革，落实居住证制度，进一步放宽入户门槛。推进事业单位人事制度改革。开展第四轮行政审批事项清理，完善推广集中审批、并联审批、网上审批。抓好石龙、塘厦扩权强镇试点，在总结经验基础上全面铺开。在镇街尝试设立政府法制机构，提高镇一级依法行政水平。探索推进黄江、厚街村级体制改革试点工作。

        深化公共事业改革。推进市政管理改革，制定精细化管理的标准和考核办法，探索实行市区数字化城管，全面推广市政设施和环卫绿化“市场化＋监理制”模式。推进水务一体化管理改革，提高供水质量。坚持公益方向，推进医疗卫生体制改革，开展公立医院改革试点。深化民政改革发展，履行部省协议，全面推进8大方面60项试点工作。深化经营性文化事业单位改革，支持民办公益性文化机构发展。完成林权改革试点。

        深化区域合作发展。深入推进深莞惠一体化发展，做好规划编制衔接，积极推动道路连接、跨界公交、收费站年票互认等工作，加强治安、治污、劳动保障、市场监管等方面的对接。深化与港台地区的经贸合作。落实产业和劳动力双转移战略，积极推进莞韶、莞惠产业转移园开发建设。加快映秀镇对口援建，确保5月12日前基本完成整体援建任务。继续帮扶河池等地区，推进与云浮、韶关两市“规划到户、责任到人”扶贫开发。

        深化机关作风建设。深入实施改进机关作风六项制度，切实提高行政效率和服务质量。弘扬艰苦奋斗，厉行勤俭节约，继续严控一般性行政经费支出，强化财政资金绩效评价，提高资金使用效率。扎实推进依法行政和廉政建设，落实重大行政决策程序和政务公开制度，主动接受市人大、市政协及社会各界监督。加大督查督办力度，以时不我待的强烈责任、以奋发有为的精神状态，以令行禁止的纪律观念，以脚踏实地的工作作风，不折不扣落实各项工作部署，努力建设“为民、务实、清廉”的服务型政府。

        各位代表！过去一年，我们众志成城、风雨同舟，经受了国际金融危机的考验，取得了极其不易的成绩。新的一年，让我们再接再厉，团结奋进，扎实做好各项工作，为推进经济社会双转型、建设富强和谐新东莞而努力奋斗！
